# Historical Legacies in World Amphibian Diversity Revealed by the Turnover and Nestedness Components of Beta Diversity

**DOI:** 10.1371/journal.pone.0032341

**Published:** 2012-02-23

**Authors:** Andrés Baselga, Carola Gómez-Rodríguez, Jorge M. Lobo

**Affiliations:** 1 Departamento de Zoología, Facultad de Biología, Universidad de Santiago de Compostela, Santiago de Compostela, Spain; 2 Departamento de Biodiversidad y Biología Evolutiva, Museo Nacional de Ciencias Naturales, CSIC, Madrid, Spain; Michigan State University, United States of America

## Abstract

Historic processes are expected to influence present diversity patterns in combination with contemporary environmental factors. We hypothesise that the joint use of beta diversity partitioning methods and a threshold-based approach may help reveal the effect of large-scale historic processes on present biodiversity. We partitioned intra-regional beta diversity into its turnover (differences in composition caused by species replacements) and nestedness-resultant (differences in species composition caused by species losses) components. We used piecewise regressions to show that, for amphibian beta diversity, two different world regions can be distinguished. Below parallel 37, beta diversity is dominated by turnover, while above parallel 37, beta diversity is dominated by nestedness. Notably, these regions are revealed when the piecewise regression method is applied to the relationship between latitude and the difference between the Last Glacial Maximum (LGM) and the present temperature but not when present energy-water factors are analysed. When this threshold effect of historic climatic change is partialled out, current energy-water variables become more relevant to the nestedness-resultant dissimilarity patterns, while mountainous areas are associated with higher spatial turnover. This result suggests that nested patterns are caused by species losses that are determined by physiological constraints, whereas turnover is associated with speciation and/or Pleistocene refugia. Thus, the new threshold-based view may help reveal the role of historic factors in shaping present amphibian beta diversity patterns.

## Introduction

The assessment of the relative roles of present and historic processes in shaping present biodiversity patterns is a central problem in macroecology [1]. The “contemporary climate” hypothesis proposes that the current availability of resources (mostly energy and water) controls the number of individuals that can live in a given area and consequently the number of species that can co-exist [2], [3]. Alternatively, the “historic” hypothesis proposes that species distributions, and thus biodiversity patterns, are controlled by past long-term processes. Such past processes are related to the contingent history of the area and/or the taxa, such as climatic stability or ancient large-scale environmental changes that may have affected species richness and composition by promoting different migration, extinction and speciation rates [4], [5]. Arguably, both contemporary and historic processes could have roles in determining present species distributions; thus, one of the key problems in macroecology is the assessment of their relative importance.

To address this question, many studies have analysed spatial variation in species richness [6], [7], [8] and, to a lesser extent, the patterns of spatial variation in composition [9], [10], [11], differentiating characteristics of populations [12] and variation in beta diversity [13], [14], [15]. The latitudinal richness gradient is probably the best-known and most-studied macroecological pattern [16], and the strong correlation of this pattern with present climatic conditions has made the “contemporary climate” hypothesis the most widely accepted explanation [2], [3], [17]. However, as climatic factors are strongly spatially structured, recent work has suggested that the climate-richness relationship, rather than being causal, could be the result of another spatially structured process [18], [19], [20]. In this context, some authors have also acknowledged the relevance of historic processes in structuring present diversity gradients [21], [22], [23]; in particular, these authors have recognised the effect of Pleistocene climatic changes on current species richness [24], [25], [26].

Historic processes may have been more important in structuring diversity patterns than it has been already proven. Some recent studies [13], [27] have shown that the examination of biodiversity measures that are more complex than species richness (e.g., variation in species composition, also called beta diversity) opens new avenues by which the relative roles of contemporary and historical factors can be assessed. The rationale here is that biotic composition contains much more information on the evolutionary processes underlying diversity patterns because it preserves the identities of species. This view is supported by studies that assess beta diversity, the results of which have suggested that a combination of present climatic and historic factors have influenced present patterns [10], [15], [28]. Despite the potential of beta diversity studies, analyses of pairwise compositional differences between assemblages at very large geographical extent (i.e. worldwide) may lack analytical power. This weakness emerges because there would be complete biotic dissimilarity (i.e. there would be no species in common) between pairs of sites that are very distant in space. Thus, if the objective is to assess whether historical events influence biotic patterns at very large scales, an alternative and feasible approach would be to define regions, calculate beta diversity and analyse the spatial variation in beta diversity among regions.

Several analyses have already addressed patterns of variation in beta diversity [29], [30], [31], suggesting that topographic heterogeneity is one of the major correlates of beta diversity within regions, followed by current climatic factors (which are mostly related to energy availability). In general, the role of historic processes (i.e. past instabilities associated with climatic changes) in shaping such patterns has only been considered indirectly or non-explicitly, but see [13], [27], [32]. In the few studies that explicitly address such processes, a clear historical signature of quaternary climate oscillations is revealed by recent methodological advances in the analysis of beta diversity (i.e. partialling out spatial turnover and nestedness components [33], [34]; see the results of Leprieur et al. for world freshwater fishes [13] and Dobrovolski et al. 2011 for New World terrestrial vertebrates [27]). These results emerge because, in analysing the abiotic drivers of beta diversity, it is important to disentangle the two different phenomena that can be behind beta diversity: spatial turnover and nestedness [33], [34]. These phenomena are antithetical because nested patterns result from species loss across sites, while turnover patterns result from species replacement. Both nested and turnover patterns are forms of beta diversity, but they are drastically different in their biological consequences. For this reason, even if two regions had the same beta diversity value, it would be misleading to consider both regions to be equivalent if one area had high turnover and low nestedness, while the second had low turnover and high nestedness [33]. This fact has been recently stressed by Leprieur et al. [13], Dobrovolski et al. [27] and Hortal et al. [26], who found that the beta diversity patterns of freshwater fish worldwide, New World vertebrates and western Palaearctic dung beetles, respectively, are dominated by nested species losses in areas that were strongly affected by Pleistocene glaciations and by species replacements in areas that experienced less severe climatic changes. Along these lines, we hypothesise the existence of a geographic threshold that separates two global regions where variation in species composition is dominated by different phenomena. Such a geographic threshold would be a consequence of climate history; particularly, it would mimic the glacial history fingerprint on a global scale. In other words, the existence of a general latitudinal gradient in beta diversity that is parallel to the latitudinal richness gradient can only be properly addressed by (i) separating the two main sources of variation in composition (species replacement vs. species losses) and (ii) identifying the regions where particular phenomena dominate the beta diversity pattern.

Using this approach, we examine the global spatial patterns of variation in the intra-regional turnover and nestedness components of beta diversity for amphibians. Ecological constraints (i.e. being ectotherms and depending on water for reproduction) cause amphibians to be remarkably sensitive to the regional availability of energy and water; they are thus a suitable group by which researchers can identify the roles of past and present climatic factors in structuring broad-scale patterns of diversity. Previous studies have suggested that water availability strongly constrains amphibian richness on a global scale [35], [36], although climatic stability may also be important [24]. Notably, available evidence suggests that glacial history plays only a minor role in explaining amphibian endemism [25] and species distribution ranges [37]. Such patterns are closely linked to beta diversity because the smaller the distribution ranges, the higher the endemism and the higher the spatial turnover. To assess whether the aforementioned threshold-based approach could reveal the influence of glacial history on amphibian assemblages, we employed three stages of investigation. First, we explored the latitudinal variation in the intra-regional turnover and nestedness fractions of beta diversity and tested for a breakpoint in the relationships between latitude and turnover and between latitude and nestedness-resultant dissimilarity components. Second, we searched for similar breakpoints in the latitudinal patterns in (i) the present climatic variables and (ii) the differences between present-day and Last Glacial Maximum (LGM) climates. The aim of this analysis was to determine which factor is the main driver of beta diversity breakpoints. Third, we estimated the capacity of present and past climatic variables to explain the variation in intra-regional turnover and nestedness-resultant dissimilarities.

## Methods

### Data origin

**Figure 1:**
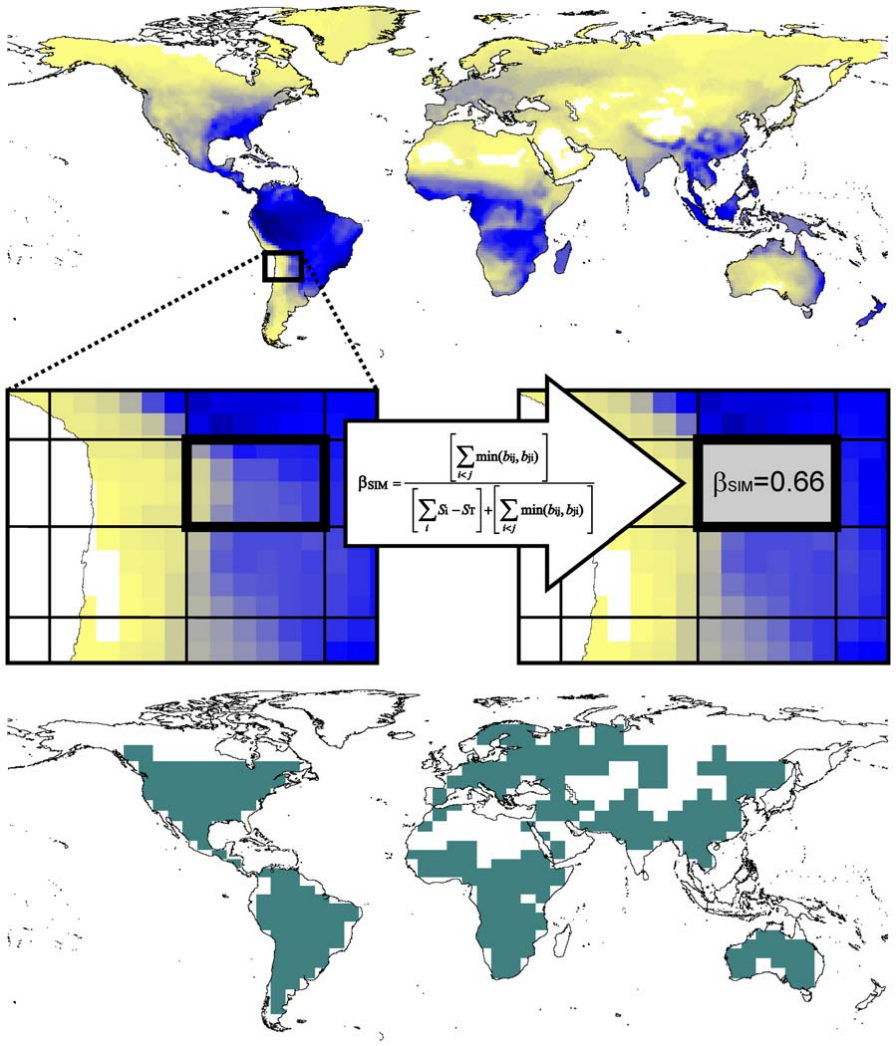
Graphical illustration of the method used to compute the multiple-site dissimilarity within regional cells of 250,000 km^2^. The original data was the presence/absence of species in 1°×1° cells (the upper map represents species richness in the 1°×1° cells, i.e. alpha diversity). A grid of regional cells of 250,000 km^2^ was superimposed on the original compositional table, and multiple-site dissimilarity was computed among all 1°×1° cells belonging to each regional cell (central row). A multiple-site dissimilarity value (β_SOR_, β_SIM_ or β_NES_) was thus assigned to each regional cell (lower map). Regional cells with a low number of 1°×1° cells (n<15) or species (gamma diversity<5) were discarded in subsequent analyses.

Global, digital amphibian range maps were obtained from expert-drawn range maps that reflect the ranges of 6157 species [38]; see data limitations in www.iucnredlist.org/. Polygonal range shapes were rasterised at a 1°×1° cell resolution. The basic units of this study are regional cells of 250,000 km^2^, which allow the measurement of beta diversity within regional cells by computing multiple-site dissimilarities among a relevant number of 1°×1° cells (Fig. 1). To define regional cells, we used the UTM grid of squares with the same area (250,000 km^2^) provided by the EDIT Geoplatform [39]. These large regional cells were superimposed on the original 1°×1° grid, and only regional cells that included more than 15 1°×1° cells and at least 5 species (regional or gamma diversity) were considered in subsequent analyses (n = 321).

Climatic data for the present and the LGM (∼21000 yr) were extracted from the ECHAM3 palaeoclimatic model [40]. The values of three contemporary variables and one past climatic variable were calculated for each terrestrial regional cell: the mean annual temperature for present time (T_PRES_), the present mean annual potential evapotranspiration (PET), the present annual precipitation (PREC) and the mean annual temperature for the LGM period (T_PAST_). The potential evapotranspiration was taken from Ahn and Tateishi [41]. The difference in average temperature between present and LGM temperatures (T_DIF_) was also calculated, as was the altitudinal variability in each regional grid cell (A_RANGE_). Altitudinal variability was calculated as the standard deviation within a regional cell, which was extracted from a global Digital Elevation Model (NASA Shuttle Radar Topography Mission: available at http://www2.jpl.nasa.gov/srtm/) with an approximate resolution of 1 km^2^ [42]. Finally, we also coded all regional cells as glaciated or non-glaciated during the LGM, following the digital cartography provided by Ehlers and Gibbard [43], [44], [45].

### Intra-regional beta diversity calculations

Intra-regional beta diversity was measured within the aforementioned regional cells of 250,000 km^2^. Intra-regional beta diversity (β_SOR_) was partitioned into its spatial turnover (β_SIM_) and nestedness (β_NES_) components, as described by Baselga [33]. In brief, this method relies on the fact that Sørensen and Simpson dissimilarities are equal in the absence of nestedness, so their difference is a measure of the nestedness component of beta diversity. Thus, for each regional cell, Sørensen multiple-site dissimilarity can be additively partitioned into two fractions, which represent spatial turnover in species composition (Simpson multiple-site dissimilarity) and variation in species composition due to nestedness (nestedness-resultant multiple-site dissimilarity). Differences in the number of 1°×1° cells among regional cells were controlled for by re-sampling 15 1°×1° cells from each regional cell 10 times and computing the average β_SOR_, β_SIM_ and β_NES_. Computations were performed in R [46] using the functions provided by Baselga [33].

### Statistical treatments

First, we assessed latitudinal gradients in intra-regional beta diversity (β_SOR_) and its turnover (β_SIM_) and nestedness (β_NES_) components; we also tested for the existence of a latitudinal breakpoint. To this end, we performed piecewise regressions between components of beta diversity and absolute latitude. We first tested all possible breakpoints at 1° intervals and selected the breakpoint that yielded the lowest residual standard error [47]. This procedure was conducted independently for β_SOR_, β_SIM_ and β_NES_. We then compared the goodness of fit of the piecewise regressions with the equivalent linear regressions by ANOVA, measuring the reduction in residual standard error. All computations were performed in R [46]. Finally, we assessed whether the existence of latitudinal breakpoints in beta diversity gradients could be connected to any (i) historic or (ii) current environmental variables. To this end, we searched for similar breakpoints in the latitudinal patterns for (i) the differences between the present and LGM temperatures (T_DIF_) and (ii) the present temperature (T_PRES_) and potential evapotranspiration (PET).

Pearson correlation coefficients using Dutilleul's [48] correction for the presence of spatial autocorrelation were used to examine the association between intra-regional β_SIM_ or β_NES_ values and environmental variables. Environmental variables significantly correlated with a given beta diversity component were included as their predictors in an ordinary least squares (OLS) regression to estimate the total percentage of variability in beta diversity that is accounted for by abiotic factors. The comparative relevance of predictors was assessed by partial regressions [49] that estimate the fractions of variation in β_SIM_ or β_NES_ that were independently explained by each predictor, as well as the shared explained variation that cannot be unambiguously attributed to any one of these predictors. The SAM v4.0 computer software (freely available at http://www.ecoevol.ufg.br/sam/) was used for all calculations [50].

## Results

### Beta diversity and richness patterns

**Figure 2:**
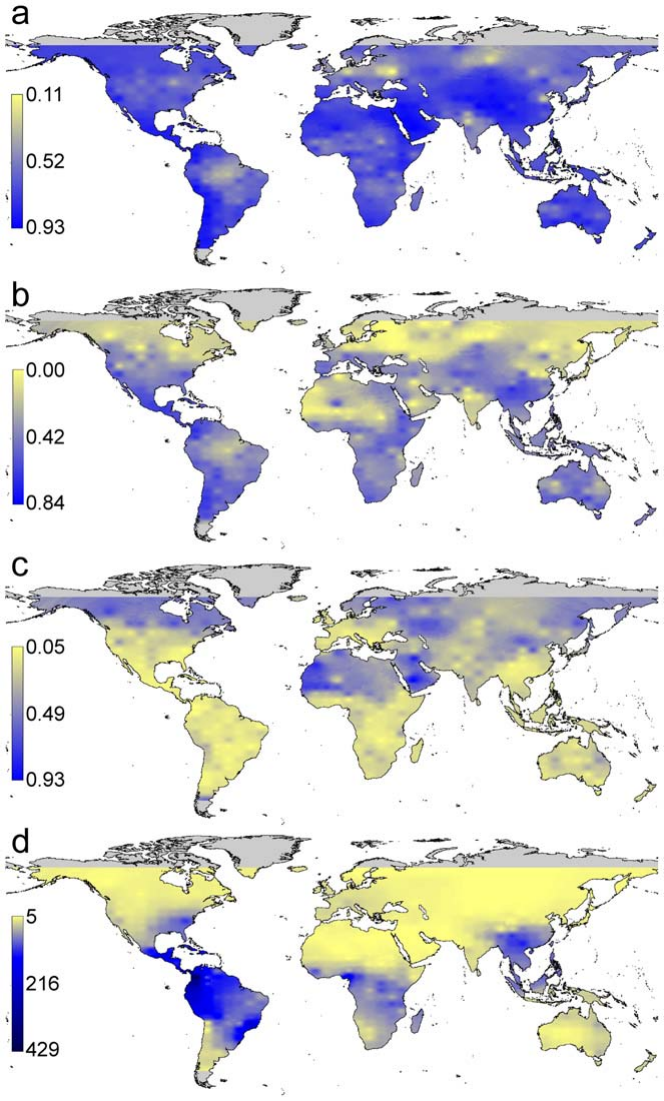
Geographic variation in the beta diversity of regional cells of 250,000 km^2^, its components and species richness: (a) beta diversity (β_SOR_), (b) spatial turnover (β_SIM_), (c) nestedness-resultant dissimilarity (β_NES_) and (d) species richness in regional cells (i.e. gamma diversity). The maps were spatially interpolated from original data points (as specified in Fig. 1) for representation by a mobile mean procedure. Colours are scaled from yellow (low values) to dark blue (high values). Grey areas represent no data.

**Figure 3:**
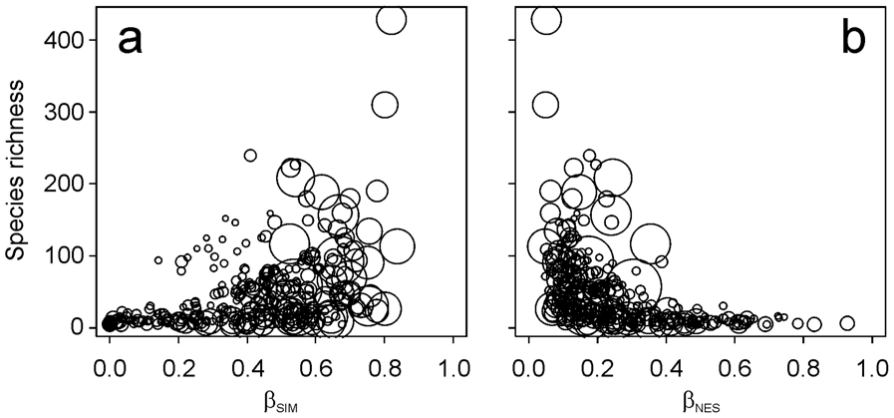
Relationship between (a) β_SIM_ and regional species richness and (b) between β_NES_ and regional species richness. Dot size is proportional to the altitudinal range of considered cells.

The world geographical pattern of amphibian β_SOR_ yielded no clear latitudinal pattern (Fig. 2a), with the lowest values in tropical and subtropical (i.e. the Amazon basin and the Indian subcontinent) and high-latitude areas (i.e. central Europe and northern Asia). In contrast, when the turnover and nestedness components were separated, clear gradients were revealed. Spatial turnover (β_SIM_) was generally higher at lower latitudes, with the exception of the Amazon basin and arid zones, such as the Sahara desert (Fig. 2b); nestedness-resultant dissimilarity (β_NES_), however, was generally higher at higher latitudes and in arid zones. These patterns were only partially related to species-richness patterns (Fig. 2d). A clear positive and significant correlation was found between regional species richness and β_SIM_ (Spearman rho = 0.55, p<0.001); consequently, a negative relationship was found between species richness and β_NES_ (rho = −0.66, p<0.001) (Fig. 3). These results suggest that, in general, a regional assemblage could not attain high species richness without high turnover and low nestedness-resultant dissimilarity. The opposite, however, was not always true because some comparatively species poor regional assemblages, which were generally associated with cells of great altitudinal heterogeneity (i.e. the southern Andes, the Rocky Mountains or the Himalayas, Fig 2), showed high turnover and low nestedness-dissimilarity values (Fig. 3).

### Latitudinal patterns of beta diversity

**Figure 4:**
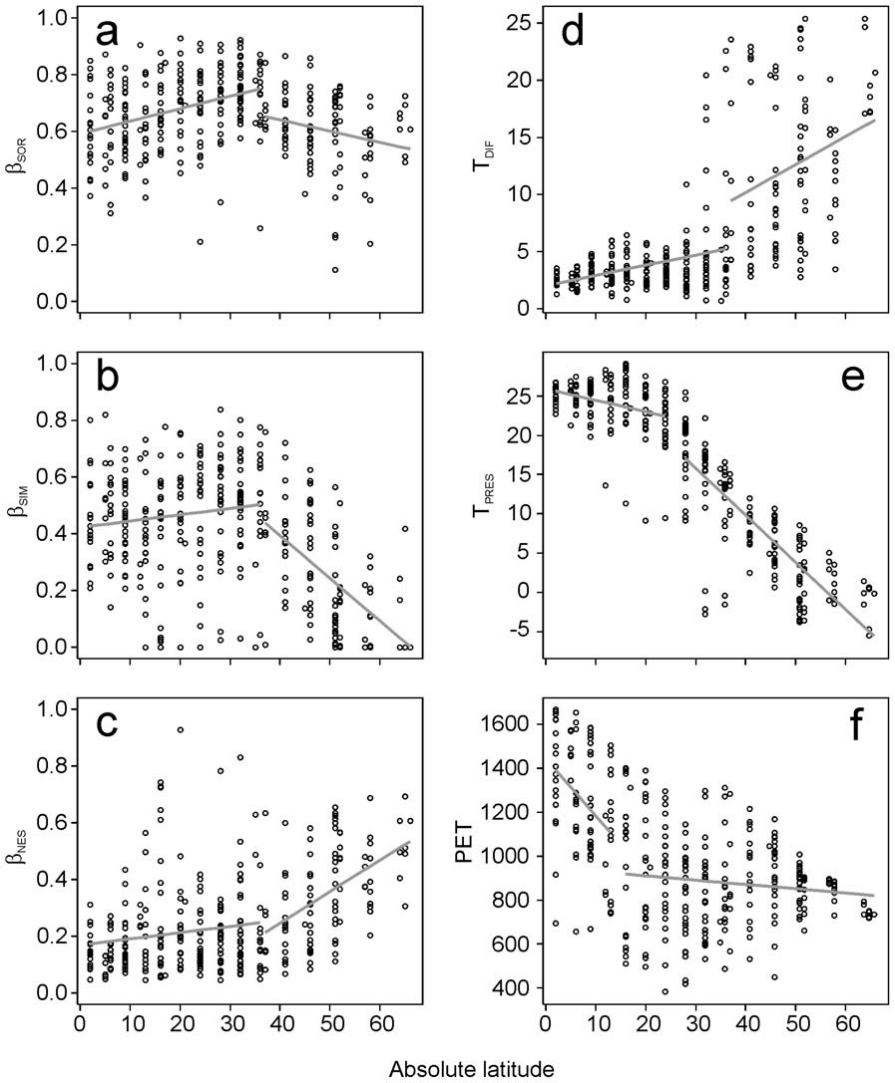
Latitudinal patterns of beta diversity and its potential explanatory variables: (a) total dissimilarity in assemblage composition (β_SOR_), (b) spatial turnover (β_SIM_), (c) nestedness-resultant dissimilarity (β_NES_), (d) temperature difference between LGM and the present conditions (T_DIF_), (e) current temperature (T_PRES_) and (f) current potential evapotranspiration (PET). Fitted functions of piecewise regression are shown (gray lines).

**Figure 5:**
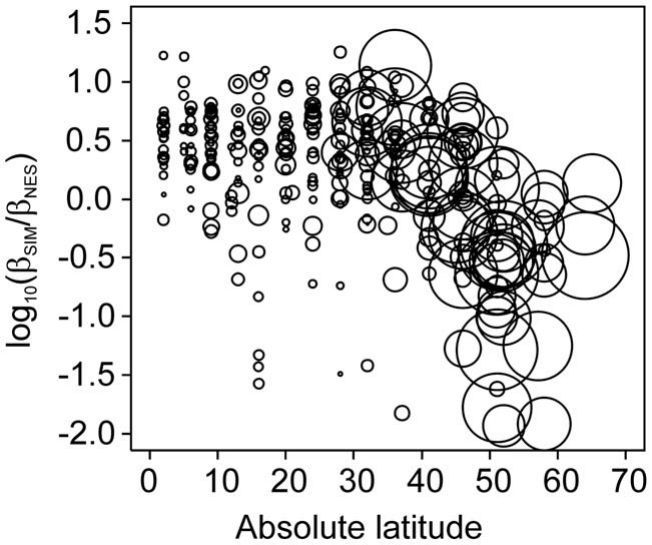
World latitudinal variation (latitude in absolute values) in the ratio between intra-regional turnover (β_SIM_) and nestedness (β_NES_) (logarithmic scale). The sizes of the circles are proportional to the difference in temperature between the present and LGM scenarios. Note the change in both the sizes of the circles and the preponderance of nestedness patterns between 30 and 40 degrees of latitude.

Piecewise regressions of β_SOR_, β_SIM_ and β_NES_ against absolute latitude (Fig. 4a–c) revealed a breakpoint at 37 degrees of latitude in the relationship between intra-regional beta diversity (and its components) and latitude. In all cases, piecewise regressions improved the fit of the model compared to the simple linear regressions: *r* ^2^ increased from 0.006 to 0.14 for β_SOR_ (*F*_2, 317_ = 25.5, p<0.001), from 0.16 to 0.30 for β_SIM_ (*F*_2, 317_ = 31.5, p<0.001) and from 0.19 to 0.23 for β_NES_ (*F*_2, 317_ = 8.0, p<0.001). In the case of β_SOR_, the slopes of the data above and below 37 degrees of latitude are not only significantly different but also have different signs (−0.003 and 0.004; *F*_2, 317_ = 14.5, p<0.001). Similarly, the breakpoint for β_SIM_ marked a transition from a shallow positive relationship (slope = 0.002) below the 37^th^ parallel to a steep negative relationship (slope = −0.016) above it (*F*_2, 317_ = 17.4, p<0.001). A lack of overlap in confidence intervals demonstrated that the mean β_SIM_ in the cells was significantly higher below 37 degrees of latitude (mean ±95% confidence interval; 0.46±0.02) than above the 37^th^ parallel (0.24±0.04). In the case of β_NES_, the regression slope changed from shallowly positive (0.002) below 37 degrees latitude to steeply positive (0.011) above the 37^th^ parallel (*F*_2, 317_ = 17.0, p<0.001). Mean β_NES_ was significantly lower in cells below 37 degrees of latitude (0.21±0.02; n = 229) than in cells above this breakpoint (0.36±0.03; n = 92). These results show that β_SIM_ and β_NES_ had contrasting relationships with latitude; compositional variation above parallel 37 was dominated by nested species losses (leading to high nestedness-resultant dissimilarity), while species replacements (leading to high spatial turnover) were the rule below parallel 37 (Fig. 5).

This pattern was mostly driven by the species in the order Anura (more than 80% of total species), as revealed by analogous analyses performed separately for the three amphibian orders (Anura, Caudata and Gymnophiona; see Appendix S1 and Fig. S1). In the case of Caudata, a similar latitudinal breakpoint was observed, although the piecewise regression models were not significantly better than the linear regressions. In Gymnophiona, all species of which were distributed below the 35^th^ parallel, no significant relationship between the beta diversity components and latitude were observed, a result consistent with the global pattern (i.e. a breakpoint around the 37^th^ parallel).

Piecewise regressions between absolute latitude and the considered climatic variables (T_DIF_, T_PRES_ and PET) also significantly improved model fit over a simple linear regression: T_DIF_ *r* ^2^ increased from 0.43 to 0.49 (*F*_2, 317_ = 18.58, p<0.01); T_PRES_ *r* ^2^ increased from 0.81 to 0.85 (*F*_2, 317_ = 42.31, p<0.01), and PET *r* ^2^ increased from 0.24 to 0.37 (*F*_2, 317_ = 32.23, p<0.01); however, only T_DIF_ showed a breakpoint (Fig. 4d) at the 37^th^ parallel, while the breakpoints for T_PRES_ and PET are located at lower latitudes (the 26^th^ and 16^th^ parallels, respectively) (Fig. 4e–f). In the case of T_DIF_, the slope of the relationship was much steeper above the breakpoint (slope changed from 0.09 to 0.24; *F*_2, 317_ = 17.7, p<0.001). Consequently, T_DIF_ values were significantly higher above 37 degrees of latitude. Moreover, a larger variation in T_DIF_ values was also observed (below the 37^th^ parallel: 3.8±0.4; above the 37^th^ parallel: 12.5±1.4).

Finally, considering only regional cells above the 37^th^ parallel, we found that cells that had contained glaciated areas during the LGM exhibited lower β_SIM_ values (0.197±0.051) than non-glaciated cells (0.288±0.062), a difference that was statistically significant (Mann-Whitney U test = 798.5; p = 0.04; n1 = 46, n2 = 46) despite the overlap in confidence intervals. Likewise, β_NES_ was slightly but significantly higher in glaciated cells (0.393±0.050) than in non-glaciated cells (0.318±0.048) (Mann-Whitney U test = 780.0; p = 0.03).

### Associated factors

**Figure 6:**
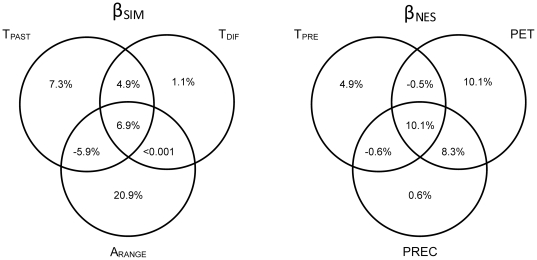
Partitioning of the variation in β_SIM_ and β_NES_ that can be explained by the significant predictors (see **Table 1**). T_PRES_ = Mean annual temperature for current times; T_PAST_ = Mean annual temperature for the LGM period; T_DIF_ = Temperature difference between the current and LGM conditions; PET = Current mean annual potential evapotranspiration; PREC = Current annual precipitation; and A_RANGE_ = Current altitudinal variability.

The variables that had the highest correlation with global spatial variation in amphibian beta diversity were different for β_SIM_ and β_NES_. For β_SIM_, the major correlates were the altitudinal range, the mean temperature during the LGM period and the difference in temperature between the LGM period and the present time (Table 1). Three explanatory variables were statistically significant for β_NES_: potential evapotranspiration, annual precipitation and present mean temperature (Table 1). Full models, including all significantly correlated variables, explained approximately one-third of total variability (35.2% for β_SIM_ and 32.9% for β_NES_). Remarkably, altitudinal variability showed a strong unique contribution in explaining the turnover (20.9%), while potential evapotranspiration made the greatest unique contribution in explaining the nestedness-resultant dissimilarity (10.1%; Fig. 6).

**Table 1:** Pearson correlation coefficients between each explanatory variable and both beta-diversity measures for the complete set of world data (Total) and the regional cells above (>37^th^) and below (<37^th^) the 37^th^ parallel.

		β_SIM_	β_NES_
	Total	0.307 (0.08)	**−0.373** (0.05)
T_PRES_	>37^th^	**0.411** (0.009)	**−0.474** (0.002)
	<37^th^	**−0.323** (0.003)	0.004 (0.98)
	Total	**0.364** (0.05)	−0.377 (0.06)
T_PAST_	>37^th^	**0.437** (0.02)	**−0.435** (0.02)
	<37^th^	**−0.287** (0.008)	0.05 (0.70)
	Total	**−0.358** (0.05)	0.283 (0.15)
T_DIF_	>37^th^	−0.269 (0.22)	0.225 (0.23)
	<37^th^	0.064 (0.53)	−0.103 (0.44)
	Total	0.259 (0.12)	**−0.529** (0.003)
PET	>37^th^	0.194 (0.16)	**−0.504** (<0.001)
	<37^th^	0.181 (0.19)	**−0.520** (0.005)
	Total	0.249 (0.08)	**−0.429** (0.007)
PREC	>37^th^	0.128 (0.46)	−0.223 (0.15)
	<37^th^	0.114 (0.31)	**−0.395** (0.009)
	Total	**0.467** (<0.001)	−0.154 (0.16)
A_RANGE_	>37^th^	**0.562** (<0.001)	−0.325 (0.08)
	<37^th^	**0.446** (<0.001)	−0.056 (0.65)

Bold values are statistically significant (p<0.05). The associated probabilities following Dutilleul's (1993) correction for the presence of spatial autocorrelation are shown in brackets. T_PRES_ = Mean annual temperature for the present; T_PAST_ = Mean annual temperature for the LGM period; T_DIF_ = Temperature difference between the present and LGM times; PET = Current annual potential evapotranspiration; PREC = Current annual precipitation; and A_RANGE_ = Altitudinal variability.

When environmental correlates were analysed separately for both of the areas identified by the piecewise regressions (i.e. above and below the 37^th^ parallel), different sets of variables were found to be significant predictors of regional patterns (Table 2). The set of variables associated with β_SIM_ were nearly the same as the variables in the global pattern, but the difference in temperature between the past and the present (T_DIF_) was substituted by the present mean temperature (T_PRES_). This set of variables (T_PRES_, T_PAST_ and A_RANGE_) was the same above and below the 37^th^ parallel (Table 1), although the signs of the correlations for temperature variables were negative below parallel 37^th^. Notably, A_RANGE_ was the predictor with the highest explanatory capacity (Table 2). Similarly, in the case of β_NES_, the set of relevant variables was nearly the same as the variables in the global pattern; the exception was annual precipitation, which was substituted by past LGM temperatures in the region above the 37^th^ parallel (Table 1). Below the 37^th^ parallel, present mean temperature had no significant effect on β_NES_.

**Table 2:** Percentage of variability (%var) explained by the variables that are significantly correlated with β_SIM_ or β_NES_ (see Table 1) for the regional cells above (>37^th^) and below (<37^th^) the 37^th^ parallel.

	β_SIM_	% var	β_NES_	% var
	Complete model	41.7	Complete model	44.6
>37°	A_RANGE_	31.5	PET	25.4
	A_RANGE_ Single	18.9	PET Single	18.4
	T_PRES_+T_PAST_	22.8	T_PRES_+T_PAST_	26.2
	T_PRES_+T_PAST_ Single	10.2	T_PRES_+T_PAST_ Single	19.2
	Shared	12.6	Shared	7.1
	Complete model	21.7	Complete model	27.4
<37°	A_RANGE_	19.9	PET	27.0
	A_RANGE_ Single	11.3	PET Single	11.8
	T_PRES_+T_PAST_	10.4	PREC	15.6
	T_PRES_+T_PAST_ Single	1.8	PREC Single	0.4
	Shared	8.6	Shared	15.2

“Complete” is the joint variability accounted for by all of the considered predictors. “Single” is the variability accounted for by a variable or group of variables after the effect of the other considered predictors has been controlled for. “Shared” is the variability that cannot be unequivocally attributed to any one of the predictors. T_PRES_ = Mean annual temperature for the present; T_PAST_ = Mean annual temperature for the LGM period; PET = Current annual potential evapotranspiration; PREC = Current annual precipitation; and A_RANGE_ = Altitudinal variability.

## Discussion

The coincidence in the latitudinal breakpoints suggests that climatic change between the present and the LGM is critical to understanding present-day patterns in beta diversity. Despite the widespread association between large-scale biodiversity patterns and the present climate [2], [3], some authors have also acknowledged that historic climate is relevant to present diversity gradients [13], [19], [22], [25]; however, it has not been easy to unravel the roles of present and past climatic variables because these variables are usually highly correlated [51]. A similar argument has been made regarding climatic stability [24]. To our knowledge, this is the first study showing that the effects of past climatic changes on global patterns of beta diversity are revealed when the influence of the past climate on present assemblages is analysed as a threshold-based effect rather than a linear and homogeneous relationship. The need for a threshold-based approach was also suggested, although not explicitly, when the stationarity of the relationship between species richness and climatic stability was analysed for dung beetles [26]. Here, we show that not only the relationship between beta diversity and latitude is non-stationary but that this characteristic is also directly linked to past climate change.

Climate conditions during Pleistocene cycles changed abruptly above a latitudinal breakpoint that roughly coincides with the 37^th^ parallel. Likewise, the present global patterns of amphibian beta diversity are characterised by a breakpoint around the 37^th^ parallel. In the area that experienced a greater change in temperature and the “bulldozer effect” of Pleistocene glaciations (above the 37^th^ parallel or, for simplicity, in “unstable areas”), the patterns in amphibian beta diversity are different from those observed in the region that experienced comparatively less dramatic climate change (below parallel 37 or in “stable areas”). Notably, when the effect of this threshold is partialled out and the analyses are conducted within each region, the historic legacy of climate is not readily apparent. For this reason, climatic stability must be analysed as a driving force of biodiversity on a very large scale because smaller scaled studies would not necessarily uncover the fingerprints of climate stability.

Our results show that beta diversity of amphibian assemblages in “unstable areas” (i.e. areas above the 37^th^ parallel) is dominated by nested species losses that lead to high nestedness-resultant dissimilarity, while species replacements that lead to high spatial turnover are the rule in “stable areas” (i.e. areas below the 37^th^ parallel). The only exceptions are desert areas at low latitudes (the Sahara and the Middle East), where aridity leads to nested species losses and thus to higher values of nestedness-resultant dissimilarity. Interestingly, the latitudinal variation of the two beta diversity components is associated in “stable areas” but unrelated in “unstable areas”; both intra-regional species turnover and nestedness-resultant dissimilarity increase with latitude in warm areas. In contrast, species turnover decreases and species nestedness increases with latitude in cold areas. Given the substantial sensitivity of amphibian species to temperature [52], the pattern seen in the unstable areas may be explained by the large “defaunation” event that resulted from the appearance of polar ice sheets and alpine glaciers [23], [51], [53] or, more generally, from very low temperatures. The subsequent colonisation of terrestrial areas after glacial retreat would create a strong nestedness pattern in a group with limited dispersal capacity. Thus, a species loss towards the north would reflect the differences in the dispersal capacity (and hence colonisation) of species after glacial retreat. The differences in intra-regional turnover and nestedness values between glaciated and non-glaciated cells in the unstable areas support this hypothesis. In contrast, the low-latitude area has experienced less dramatic climatic change, a fact that would have favoured the long-term persistence of assemblages. Species assemblages at low latitudes would therefore be older than assemblages in high-latitude regions [22], a pattern that is in agreement with the “climatic stability” hypothesis [53], [54]. Species turnover would be expected to be higher in areas with older assemblages because speciation events accumulate over time. This accumulation would result in new species with smaller ranges than ancestral species because evolutionary processes (i.e. speciation and extinction events) gain relevance as the drivers of assemblage composition with time. For amphibians, diversification rates have been shown to increase with decreasing latitude [55]. This major pattern in beta diversity is in accordance with patterns reported for freshwater fishes [13], New World vertebrates [27] and European longhorn beetles [33]. Therefore, contrasting patterns of beta diversity between high and low latitudes appear to be a general rule for many taxa.

The general pattern is mostly driven by Anura, while Gymnophiona and Caudata represent special cases. In the case of Gymnophiona, all species are restricted to latitudes below the 35^th^ parallel (i.e. stable areas), so the lack of significant relationships between beta diversity components and latitude is consistent with the general pattern and the climatic instability hypothesis. The case of Caudata is, in turn, slightly different because the breakpoint is detected in the same latitude but the piecewise models are not significantly better than linear regressions. This difference could be explained by the temperate origin and the thermal requirements of Caudata. Most Caudata species have typical activity temperatures below 15°C, even in the tropics [52], which restricts tropical Caudata (Plethodontids) to the mountains. As a consequence, tropical Caudata have small, fragmented distribution ranges, thus increasing spatial turnover to the equator. In parallel, the temperate origin of Caudata implies that large areas below the 37^th^ parallel have very few urodelan species. As a consequence, the number of cells used in the analysis is comparatively lower for Caudata than for Anura. This lower number of cells makes more difficult to obtain significant values in the piecewise regressions. In sum, despite the probable higher relevance of current temperature for Caudata, the three orders present patterns in agreement with the hypothesis that historical climatic instability had a preeminent role in shaping beta diversity.

Once the role of climatic instability is partialled out, independent explanations for the roles of climatic and topographic factors in structuring beta-diversity patterns may apply in each area. Moreover, because alternative processes (i.e. species losses *vs.* species replacements) may generate variations in beta diversity, it is also important to distinguish between the effects of climatic and topographic variables in the two components of beta diversity: turnover and nestedness-resultant dissimilarity. If both components were combined in a single measure of beta diversity (i.e. the popular Whittaker, Sørensen and Jaccard indices or the additive beta diversity), we would not be able to connect the beta diversity values with the different processes that lead to species losses or species replacements. Therefore, it would be difficult to assess the relationship between biotic dissimilarity and its environmental or historic drivers, as was shown in a previous study [13]. In contrast, by separating the components of beta diversity, we can link the different patterns of variation in species composition (turnover and nestedness) with their potential drivers. In the case of amphibians, once the binary effect of historic climatic change was accounted for, only present climatic variables were associated with the nestedness component of beta diversity, whereas altitudinal range appeared to be the factor that was most strongly correlated with turnover. Nestedness-resultant dissimilarity seems to be higher when present values of evapotranspiration, precipitation and temperature are low, and the relevance of these variables is higher in unstable and cold areas. In particular, the strong effect of evapotranspiration suggests that water availability is critical for explaining global patterns of amphibian beta diversity. This factor also significantly influences richness patterns [35], [36]. Thus, in areas where water availability is a limiting factor (i.e. arid deserts), species disappear sequentially from the assemblages because of their physiological constraints, creating a nested pattern that is mostly responsible for the beta diversity of such regions. Although energy-related variables are secondary factors, their effect on intra-regional turnover is remarkable because these variables have opposing effects in stable and unstable areas. Intra-regional turnover is negatively correlated with temperature in the stable areas but positively correlated with temperature in the unstable areas. This pattern suggests a non-causal relationship between temperature and turnover or, alternatively, a unimodal relationship with higher turnover corresponding to intermediate temperatures.

It is frequently claimed that climate- and energy-related variables have causal relationships with present diversity gradients at regional or global scales [56]. Previous studies on beta diversity have suggested that climatic variables are associated with the turnover of European mammals [15] as well as turnover of birds and amphibians on a global scale [57]. Regarding amphibian diversity patterns, topographic variability (elevation range) does not seem to be a major contributor to global amphibian richness [36]. Given these previous contributions, it is remarkable that we have found altitudinal range to be important in structuring amphibian beta diversity. Our results suggest that mountains could have profoundly influenced present intra-regional turnover in amphibian composition, with greater significant impacts than the present temperature in both warm and cold areas. Some authors [58] suggest that the role of altitudinal range should be viewed as an indirect effect of climate. From the same perspective, altitudinal range is considered to be a proxy for habitat or climatic heterogeneity (i.e. mountain areas harbour a wide gradient of environmental conditions over a short distance); in turn, these conditions would favour species diversity through niche filtering. Alternatively, an historic explanation is also plausible because mountainous areas may act as refuges, facilitating the persistence of some species [59] and the emergence of evolutionary novelties [60], [61] by favouring allopatric speciation and the occurrence of new adaptations [62]. The glacial refuge hypothesis may be important for species with limited dispersal capacity, such as amphibians [63]; these species could have survived climatic oscillations by tracking climate change across short distances.

Finally, it is necessary to acknowledge some limitations of our analyses. Our dataset was derived from expert-drawn global range maps for amphibians [38]. These maps were converted into presence/absence matrices in 1°×1° cells, and the measures of beta diversity (turnover and nestedness-based dissimilarity) were computed for larger regional cells of 250,000 km^2^. First, range maps are idealised distributions that interpolate continuous species ranges based on discrete known presence records. This process could make our estimates of beta diversity lower than they should be, as gaps within real distributions are not mirrored by range maps. Given the scale of our analysis (1°×1° cells), however, this bias is likely limited; the probability of finding gaps in species distributions at this spatial resolution is low. Second, this scale of analysis has its own limitations, as we ignore the compositional heterogeneity occurring on smaller scales. However, the loss of information due to the coarse resolution is compensated for by the increase in robustness, as distributional data at smaller spatial scales would be more affected by temporal dynamics and stochasticity. Finally, amphibians are not a hyperdiverse group, and a number of regional cells had to be excluded from analyses because they contained an extremely low number of species. Beta-diversity measures depend on the proportion of species that are replaced (or lost) between cells, so they are prone to marked stochastic variation when the species number is very low; under these conditions, the replacement of only one species would substantially influence beta diversity. We therefore excluded large areas from our analyses, but as previously stated, this loss of information is compensated for, in our opinion, by the increased robustness of the dataset.

### Conclusions

Global patterns of amphibian beta diversity suggest that present biodiversity has likely been affected by climatic changes that have occurred since the Pleistocene. This correlation, however, was revealed only by a threshold-based approach. This approach allowed us to estimate the inflection point in the relationship between intra-regional beta diversity components and latitude. Thus, the correspondence of such latitudinal breakpoint with breakpoints in potential explanatory factors could be assessed. Our results show that beta diversity and the temperature difference between the present and the LGM show similar, non-linear, latitudinal patterns that define two global regions; spatial turnover dominates the pattern of assemblage variation in the climatically “stable areas”, whereas nested patterns dominate the “unstable areas”. When this threshold effect is partialled out, the relevance of the present climatic and energetic variables to the intra-regional nestedness dissimilarity patterns becomes evident, and mountains are clearly associated with higher spatial turnover. This result suggests that nested patterns are caused by species losses determined by physiological constraints, whereas turnover seems to be associated with speciation and the existence of Pleistocene refugia. As a final consideration, we believe that the preeminence of contemporary climatic-energetic factors as explanation for biodiversity gradients results from a failure to consider the binary role of climatic stability on a global scale. Thus, our novel approach contributes to the debate on the relative importance of historic and contemporary factors in shaping present biodiversity patterns, supporting the primacy of historic factors.

## Supporting Information

Appendix S1 Latitudinal patterns of the beta-diversity components in the three amphibian orders (Anura, Caudata and Gymnophiona). Figure S1 Latitudinal patterns of beta diversity componens (β_SIM_ and β_NES_) in Anura (a, b), Caudata (c, d) and Gymnophiona (e, f). Fitted functions are shown: piecewise regressions (blue), linear regressions (red).
(DOC)

## References

[1] Ricklefs RE (2004). A comprehensive framework for global patterns in biodiversity.. Ecology Letters.

[2] Currie DJ, Mittelbach GG, Cornell HV, Field R, Guegan J-F (2004). Predictions and tests of climate-based hypotheses of broad-scale variation in taxonomic richness.. Ecology Letters.

[3] Hawkins BA, Field R, Cornell HV, Currie DJ, Guegan JF (2003). Energy, water, and broad-scale geographic patterns of species richness.. Ecology.

[4] Dynesius M, Jansson R (2000). Evolutionary consequences of changes in species' geographical distributions driven by Milankovitch climate oscillations.. Proceedings of the National Academy of Sciences of the United States of America.

[5] Ricklefs RE (2006). Evolutionary diversification and the origin of the diversity-environment relationship.. Ecology.

[6] Hawkins BA, Porter EE (2003). Relative influences of current and historical factors on mammal and bird diversity patterns in deglaciated North America.. Global Ecology and Biogeography.

[7] Kerr JT, Currie DJ (1999). The relative importance of evolutionary and environmental controls on broad-scale patterns of species richness in North America.. Ecoscience.

[8] Ricklefs RE, Latham RE, Qian H (1999). Global patterns of tree species richness in moist forests: distinguishing ecological influences and historical contingency.. Oikos.

[9] Latham RE, Ricklefs RE, Ricklefs RE, Schluter D (1993). Continental comparisons of temperate-zone tree species diversity.. Species diversity in ecological communities: historical and geographical perspectives.

[10] Svenning JC, Kinner DA, Stallard RF, Engelbrecht BMJ, Wright SJ (2004). Ecological determinism in plant community structure across a tropical forest landscape.. Ecology.

[11] Zobel M, Otto R, Laanisto L, Naranjo-Cigala A, Pärtel M (2011). The formation of species pools: historical habitat abundance affects current local diversity.. Global Ecology and Biogeography.

[12] Heaney LR, Walsh JS, Peterson AT (2005). The roles of geological history and colonization abilities in genetic differentiation between mammalian populations in the Philippine archipelago.. Journal of Biogeography.

[13] Leprieur F, Tedesco PA, Hugueny B, Beauchard O, Dürr HH (2011). Partitioning global patterns of freshwater fish beta diversity reveals contrasting signatures of past climate changes.. Ecology Letters.

[14] Melo AS, Rangel TFLVB, Diniz-Filho JAF (2009). Environmental drivers of beta-diversity patterns in New-World birds and mammals.. Ecography.

[15] Svenning JC, Fløjgaard C, Baselga A (2011). Climate, history and neutrality as drivers of mammal beta diversity in Europe: insights from multiscale deconstruction.. Journal of Animal Ecology.

[16] Willig MR, Kaufman DM, Stevens RD (2003). Latitudinal gradients of biodiversity: Pattern, process, scale, and synthesis.. Annual Review of Ecology Evolution and Systematics.

[17] Whittaker RJ, Nogués-Bravo D, Araújo MB (2007). Geographical gradients of species richness: a test of the water-energy conjecture of Hawkins et al. (2003) using European data for five taxa.. Global Ecology and Biogeography.

[18] Bahn V, McGill BJ (2007). Can niche-based distribution models outperform spatial interpolation?. Global Ecology and Biogeography.

[19] Svenning JC, Skov F (2007). Could the tree diversity pattern in Europe be generated by postglacial dispersal limitation?. Ecology Letters.

[20] Tello JS, Stevens RD (2011). Can stochastic geographical evolution re-create macroecological richness–environment correlations?. Global Ecology and Biogeography.

[21] Hawkins BA (2010). Multiregional comparison of the ecological and phylogenetic structure of butterfly species richness gradients.. Journal of Biogeography.

[22] Hawkins BA, Diniz-Filho JAF, Jaramillo CA, Soeller SA (2006). Post-Eocene climate change, niche conservatism, and the latitudinal diversity gradient of New World birds.. Journal of Biogeography.

[23] Montoya D, Rodríguez MA, Zavala MA, Hawkins BA (2007). Contemporary richness of holarctic trees and the historical pattern of glacial retreat.. Ecography.

[24] Araújo MB, Nogués-Bravo D, Diniz-Filho JAF, Haywood AM, Valdes PJ (2008). Quaternary climate changes explain diversity among reptiles and amphibians.. Ecography.

[25] Jansson R (2003). Global patterns in endemism explained by past climatic change.. Proceedings of the Royal Society of London Series B-Biological Sciences.

[26] Hortal J, Diniz-Filho JAF, Bini LM, Rodríguez MA, Baselga A (2011). Ice age climate, evolutionary constraints and diversity patterns of European dung beetles.. Ecology Letters.

[27] Dobrovolski R, Melo AS, Cassemiro FAS, Diniz-Filho JAF (2012). Climatic history and dispersal ability explain the relative importance of turnover and nestedness components of beta diversity.. Global Ecology and Biogeography.

[28] Baselga A (2008). Determinants of species richness, endemism and turnover in European longhorn beetles.. Ecography.

[29] Gaston KJ, Davies RG, Orme CDL, Olson VA, Thomas GH (2007). Spatial turnover in the global avifauna.. Proceedings of the Royal Society B-Biological Sciences.

[30] McKnight MW, White PS, McDonald RI, Lamoreux JF, Sechrest W (2007). Putting beta-diversity on the map: Broad-scale congruence and coincidence in the extremes.. Plos Biology.

[31] Qian H, Ricklefs RE (2007). A latitudinal gradient in large-scale beta diversity for vascular plants in North America.. Ecology Letters.

[32] Graham CH, Moritz C, Williams SE (2006). Habitat history improves prediction of biodiversity in rainforest fauna.. Proceedings of the National Academy of Sciences of the United States of America.

[33] Baselga A (2010). Partitioning the turnover and nestedness components of beta diversity.. Global Ecology and Biogeography.

[34] Baselga A (2012). The relationship between species replacement, dissimilarity derived from nestedness and nestedness.. Global Ecology and Biogeography.

[35] Buckley LB, Jetz W (2007). Environmental and historical constraints on global patterns of amphibian richness.. Proceedings of the Royal Society B-Biological Sciences.

[36] Qian H (2010). Environment-richness relationships for mammals, birds, reptiles, and amphibians at global and regional scales.. Ecological Research.

[37] Whitton FJS, Purvis A, Orme CDL, Olalla-Tarraga MA (2011). Understanding global patterns in amphibian geographic range size: does Rapoport rule?. Global Ecology and Biogeography.

[38] IUCN (2009). IUCN Red List of Threatened Species.. http://www.iucnredlist.org.

[39] Sastre P, Roca P, Lobo JM, EDIT co-workers (2009). A Geoplatform for the accessibility to environmental cartography.. Journal of Biogeography.

[40] Braconnot P, Otto-Bliesner B, Harrison S, Joussaume S, Peterchmitt JY (2007). Results of PMIP2 coupled simulations of the Mid-Holocene and Last Glacial Maximum - Part 1: experiments and large-scale features.. Climate of the Past.

[41] Ahn C-H, Tateishi R (1994). Development of a global 30-minute grid potential evapotranspiration data set.. Journal of the Japan Society of Photogrammetry and Remote Sensing.

[42] Clark Labs (2000). Gobal change data archive Vol. 3. 1 km global elevation model.

[43] Ehlers J, Gibbard PL (2004). Extent and chronology of glaciation. Volume 1: Europe.

[44] Ehlers J, Gibbard PL (2004). Extent and chronology of glaciation. Volume 2: North America.

[45] Ehlers J, Gibbard PL (2004). Extent and chronology of glaciation. Volume 3: South America, Asia, Africa, Australia, Antarctica.

[46] R Development Core Team (2009). R: A language and environment for statistical computing. Version 2.10.1. Available at http://www.r-project.org.

[47] Crawley MJ (2007). The R book.

[48] Dutilleul P (1993). Modifying the t test for assessing the correlation between two spatial processes.. Biometrics.

[49] Legendre P, Legendre L (1998). Numerical ecology, 2nd ed.

[50] Rangel TF, Diniz JAF, Bini LM (2010). SAM: a comprehensive application for spatial analysis in macroecology.. Ecography.

[51] Hawkins BA, Porter EE (2003). Water-energy balance and the geographic pattern of species richness of western Palearctic butterflies.. Ecological Entomology.

[52] Wells KD (2007). The ecology and behavior of amphibians.

[53] Fischer AG (1960). Latitudinal variations in organic diversity.. Evolution.

[54] Pianka ER (1966). Latitudinal gradients in species diversity: a review of concepts.. American Naturalist.

[55] Wiens JJ (2007). Global patterns of diversification and species richness in amphibians.. American Naturalist.

[56] Wright DH (1983). Species-energy theory - an extension of species-area theory.. Oikos.

[57] Buckley LB, Jetz W (2008). Linking global turnover of species and environments.. Proceedings of the National Academy of Sciences of the United States of America.

[58] Ruggiero A, Hawkins BA (2008). Why do mountains support so many species of birds?. Ecography.

[59] Fjeldså J, Lambin E, Mertens B (1999). Correlation between endemism and local ecoclimatic stability documented by comparing Andean bird distributions and remotely sensed land surface data.. Ecography.

[60] Ghalambor CK, Huey RB, Martin PR, Tewksbury JJ, Wang G (2006). Are mountain passes higher in the tropics? Janzen's hypothesis revisited.. Integrative and Comparative Biology.

[61] Kozak KH, Wiens JJ (2006). Does niche conservatism promote speciation? A case study in North American salamanders.. Evolution.

[62] Hoorn C, Wesselingh FP, ter Steege H, Bermudez MA, Mora A (2010). Amazonia through time: Andean uplift, climate change, landscape evolution, and biodiversity.. Science.

[63] Smith MA, Green DM (2005). Dispersal and the metapopulation paradigm in amphibian ecology and conservation: are all amphibian populations metapopulations?. Ecography.

