# Supplementary material for: Historical Legacies in World Amphibian Diversity Revealed by the Turnover and Nestedness Components of Beta Diversity
Source: PLoS One. 2012 Feb 23;7(2):e32341. doi: 10.1371/journal.pone.0032341 (PMC3285684; doi:10.1371/journal.pone.0032341)

**Appendix S1.**

**Supporting information for:**

**Historical legacies in world amphibian diversity revealed by the turnover and nestedness components of beta diversity**

Andrés Baselga^1*^, Carola Gómez-Rodríguez^1^ and Jorge M. Lobo^2^

^1^Departamento de Zoología, Facultad de Biología, Universidad de Santiago de Compostela, Rúa Lope Gómez de Marzoa s/n, 15782 Santiago de Compostela, Spain.

^2^Departmento de Biodiversidad y Biología Evolutiva, Museo Nacional de Ciencias Naturales, CSIC, C/Gutiérrez Abascal, 2, 28006 Madrid, Spain.

**Appendix S1.** Latitudinal patterns of the beta-diversity components in the three amphibian orders (Anura, Caudata and Gymnophiona).

To assess whether the latitudinal breakpoint in the gradients of beta diversity components recovered for the whole amphibian dataset could also be recovered for the three amphibian orders separately (Anura, Caudata and Gymnophiona), we performed a set of piecewise regressions for each of the three groups. In the case of Caudata and Gymnophiona, the number of species in regional cells was low. A low number of species can make beta-diversity values unstable because the absence or replacement of only one species represents a large proportion of the total species pool; however, an exigent minimum number of species would cause the exclusion of a large proportion of the regional cells. Thus, we analysed all regional cells with at least 2 species (n =114 for Caudata and n =76 for Gymnophiona). In the case of Anura, all 321 regional cells considered in the main text contained at least 3 species.

**Anura**

For the turnover component turnover (β_SIM_), the piecewise regression revealed a latitudinal breakpoint at the 35^th^ parallel (Fig. S1a). This piecewise regression (*r*^2^ =0.30) significantly increased model fit (*F*_2, 317_ = 25.1, p < 0.001) compared to the linear regression (*r*^2^ =0.20). For the nestedness component of beta diversity (β_NES_), the iterative search for the breakpoint revealed that the residual standard error was almost as low (RSS = 0.1579 – 0.1583) for models with breakpoints at the 16^th^, 22^nd^, 35^th^, and 50^th^ parallels. Among these models, we selected the 35^th^ parallel (Fig. S1b), as this model was in agreement with previous results. This piecewise regression increased model *r*^2^ from 0.17 (as in the linear regression) to 0.20 (*F*_2, 317_ = 4.6, p = 0.011).

**Caudata**

For the turnover component turnover (β_SIM_), the iterative search for the breakpoint revealed that the residual standard error was lowest for a model with the latitudinal breakpoint at the 35^th^ parallel; however, this model (*r*^2^ =0.45) did not significantly increase model fit (*F*_2, 110_ = 1.62, p = 0.202) compared to the linear regression (*r*^2^ =0.44; Fig. S1c). The same result was found for the nestedness component of beta diversity (β_NES_), for which the lowest residual standard error in the piecewise regression was detected for a breakpoint at the 35^th^ parallel. This model (*r*^2^ =0.16) did not significantly increase model fit (*F*_2, 110_ = 1.41, p = 0.249) compared to the linear regression (*r*^2^ =0.14; Fig. S1d). Therefore, β_SIM_ decreases and β_NES_ increases linearly with latitude in Caudata, although a change in the slopes of both relationships around the 35^th^ parallel is suggested by the peaks in residual standard error of the piecewise models. These peaks are not significant, likely because of the lower number of cases and the high scatter (a result of the low number of species in many cells).

**Gymnophiona**

In contrast to the previous groups, data for Gymnophiona were restricted to regional cells below parallel 35^th^. For the turnover component turnover (β_SIM_), the iterative search for the breakpoint revealed that the residual standard error was lowest for a model with the latitudinal breakpoint at the 16^th^ parallel; however, this model was not significant (*r*^2^ =0.04, *F*_3, 72_ = 0.97, p = 0.414) and did not significantly increase model fit (*F*_2, 72_ = 1.34, p = 0.269) compared to the linear regression, which was also not significant (*r*^2^<0.01, *F*_1, 74_ = 0.22, p = 0.642; Fig. S1e). The same result was found for the nestedness component of beta diversity (β_NES_), for which the lowest residual standard error in the piecewise regression was detected for a breakpoint at the 35^th^ parallel. This model (*r*^2^ =0.04, *F*_3, 72_ = 0.96, p = 0.415) was non-significant and did not significantly increase model fit (*F*_2, 110_ = 0.47, p = 0.629) compared to the linear regression, which was also not significant (*r*^2^ =0.03, *F*_1, 74_ = 1.99, p = 0.163; Fig. S1f). Therefore, both beta-diversity components showed a latitudinal gradient in Gymnophiona. This result is consistent with the general pattern, as all caecilians are distributed below the 35^th^ parallel.

**Figure S1**. Latitudinal patterns of beta diversity componens (β_SIM_ and β_NES_) in Anura (a, b), Caudata (c, d) and Gymnophiona (e, f). Fitted functions are shown: piecewise regressions (blue), linear regressions (red).


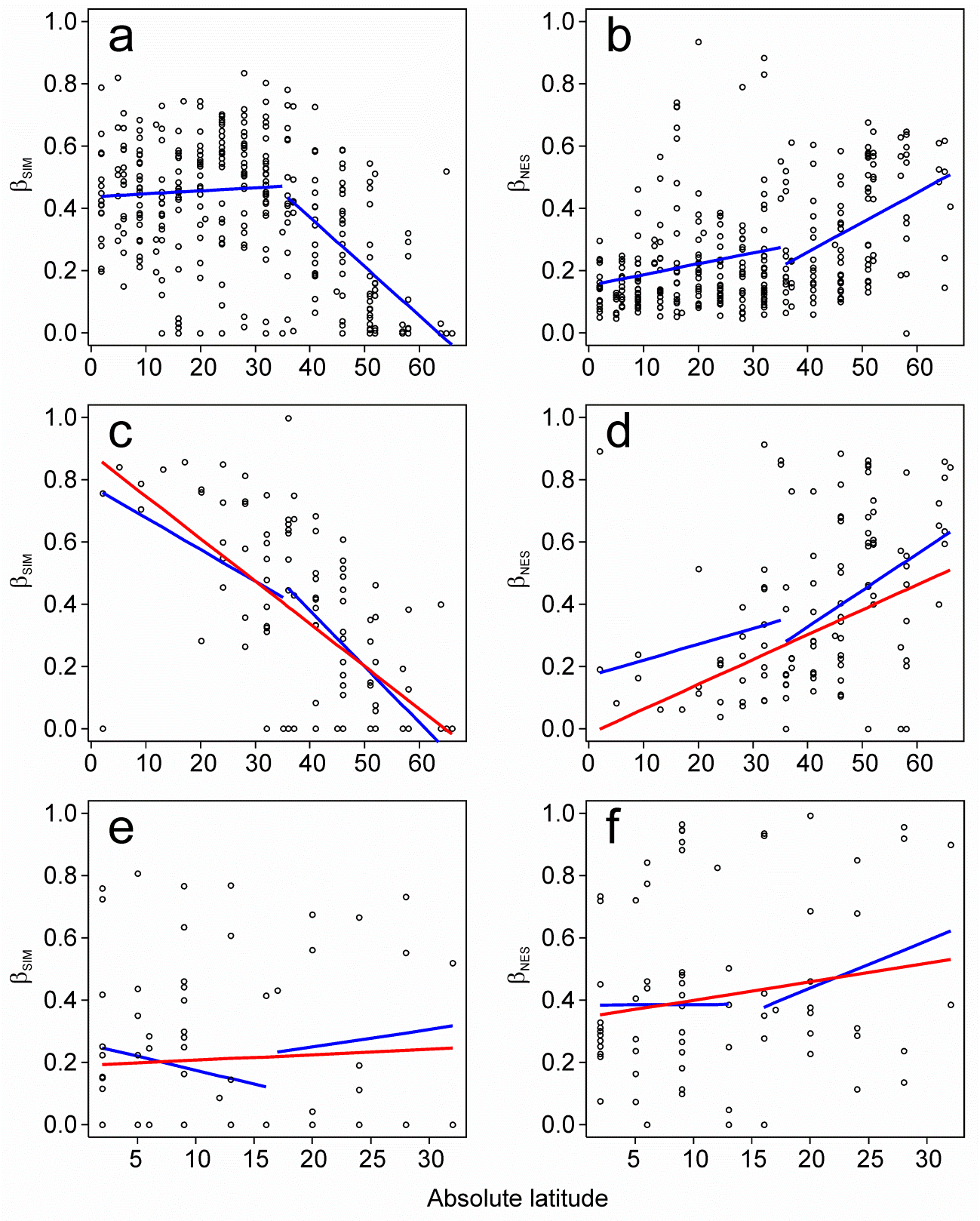

Supplement: Appendix S1 — Latitudinal patterns of the beta-diversity components in the three amphibian orders (Anura, Caudata and Gymnophiona). Figure S1 Latitudinal patterns of beta diversity componens (βSIM and βNES) in Anura (a, b), Caudata (c, d) and Gymnophiona (e, f). Fitted functions are shown: piecewise regressions (blue), linear regressions (red). (DOC) [file pone.0032341.s001.doc]
